# Dielectric Study on Supramolecular Gels by Fiber Structure Formation from Low-Molecular-Weight Gelator/Water Mixtures

**DOI:** 10.3390/gels9050408

**Published:** 2023-05-12

**Authors:** Kenta Shimizu, Fumiya Abe, Yasuhiro Kishi, Rio Kita, Naoki Shinyashiki, Shin Yagihara

**Affiliations:** 1 Graduate School of Science, Tokai University, 4-1-1 Kitakaname, Hiratsuka-shi 259-1292, Japan; dynamite724@gmail.com (K.S.); ab3212.moqt@gmail.com (F.A.); kyiivsmynp@gmail.com (Y.K.); 2 Department of Physics, School of Science, Tokai University, Hiratsuka-shi 259-1292, Japan; rkita@keyaki.cc.u-tokai.ac.jp (R.K.); naoki-ko@keyaki.cc.u-tokai.ac.jp (N.S.); 3 Micro/Nano Technology Center, Tokai University, Hiratsuka-shi 259-1292, Japan

**Keywords:** structure formation, heterogeneity, hydrogels, supramolecular gel, sol–gel transition, molecular dynamics, broadband dielectric spectroscopy

## Abstract

There are various types of gel materials used in a wide range of fields, and their gelation mechanisms are extremely diverse. Furthermore, in the case of hydrogels, there exist some difficulties in understanding complicated molecular mechanisms especially with water molecules interacting through hydrogen bonding as solvents. In the present work, the molecular mechanism of the structural formation of fibrous super-molecular gel by the low molecular weight gelator, N-oleyl lactobionamide/water mixture was elucidated using the broadband dielectric spectroscopy (BDS) method. The dynamic behaviors observed for the solute and water molecules indicated hierarchical structure formation processes in various time scales. The relaxation curves obtained at various temperatures in the cooling and heating processes showed relaxation processes respectively reflecting the dynamic behaviors of water molecules in the 10 GHz frequency region, solute molecules interacting with water in MHz region, and ion-reflecting structures of the sample and electrode in kHz region. These relaxation processes, characterized by the relaxation parameters, showed remarkable changes around the sol–gel transition temperature, 37.8 °C, determined by the falling ball method and over the temperature range, around 53 °C. The latter change suggested a structure formation of rod micelles appearing as precursors before cross-linking into the three-dimensional network of the supramolecular gels. These results clearly demonstrate how effective relaxation parameter analysis is for understanding the gelation mechanism in detail.

## 1. Introduction

Supramolecular gels are essentially important materials in modern technology, since they are applied to substance separating in chemical processes, technology for environmental cleanup, biotechnology, drug development and regenerative medicine [1]. Supramolecular gels used in industry are generally natural polymer gels, such as agarose gel, and synthetic polymer gels, such as acrylamide gel. In order to give functions to these gels, there are some drawbacks such as their complicated preparation, the presence of unreacted substances, and the difficulty of precise molecular design. In contrast, in the studies on fibrous supramolecular gels, gel structures and functions can be controlled by the appropriate design of the monomer [1,2,3]. However, in order to understand the structure and properties of these gels [4,5,6,7] and realize their applications in more diverse fields [8,9,10], we need to clarify the more essential molecular mechanisms common to all polymer gels.

**Figure 1:**
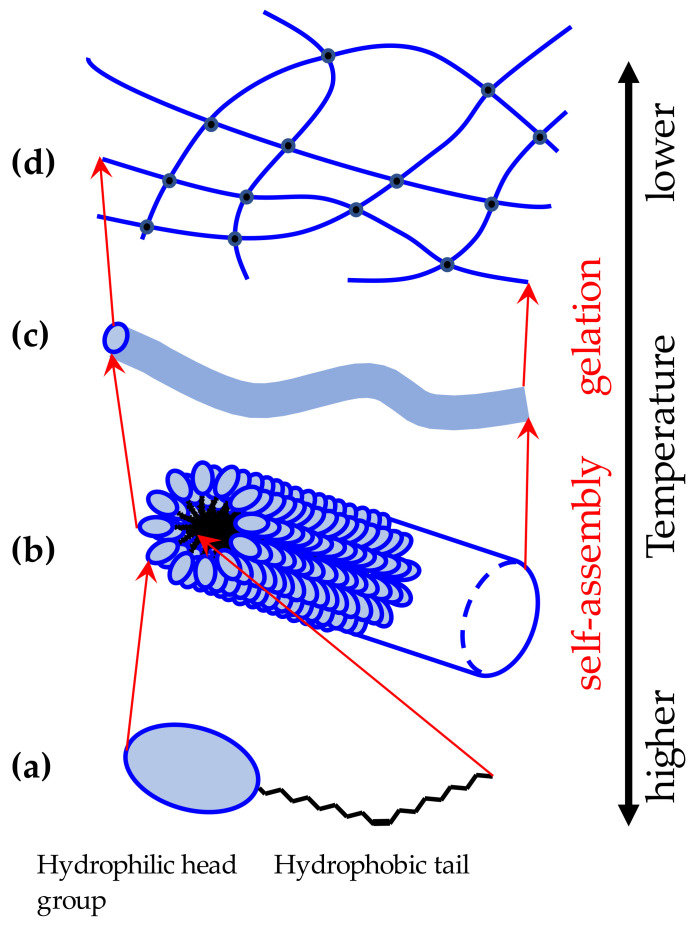
Hierarchical structures of fiber-crosslinked supramolecular gels by rod micelle growth of low-molecular-weight gelators in water: (**a**) amphiphilic molecules, (**b**) rod micelles, (**c**) fiber elongation, (**d**) fiber crosslinking.

Generally, amphiphilic compounds of low molecular weight form spherical associations and are emulsifying agents when they are dissolved in a solution. However, the low-molecular-weight hydrogelator molecules form rod micelles even though their molecular structure is also amphipathic [10], and the rod micelles are extended in one-dimensional direction by self-assembly in water to form fibers, as shown in Figure 1. Figure 1 is a schematic diagram showing an example of the hierarchical structure of a supramolecular gel that is crosslinked by the elongation of micelles formed by low-molecular-weight gelators. The conformations of the alkyl chains of the amphiphilic molecules are kept the same for convenience, and the figure is provided to show the schematic formation of rod micelles through non-covalent interactions. In this study, we focus on the transition phenomenon between the sol state at high temperature and the gel state at low temperature. Monomers in supramolecular gels exhibit several types of small aggregates, interacting with other monomers and water molecules via hydrogen bonding [11,12,13,14,15,16], but in any case, fibrous aggregates gather and entangle as a three-dimensional network structure to form hydrogels with water molecules [10]. In addition, hydrophobic domains are formed in rod micelles, and filamentous associations are formed along with their formation [17].

Since the hierarchical structure of these supramolecular gels spans a wide spatiotemporal region [18], experimental methods to observe and analyze them should also preserve their broadband properties. The observation and analysis of water molecules are also important, because the association structure of molecules in supramolecular hydrogels is caused by interactions with water molecules, and the behavior of water molecules plays a major role [11,12,13]. In general, the structure and physical properties of gels strongly depend on the temperature and water content, so the evaluation of fluctuations of structures and physical properties is very important [19].

Research on supramolecular gels has made great strides in recent years, from the development of novel gelling agents [20,21,22] and applications to advanced functions such as photoactivity [23] and interactions with solvents [24]. Fundamental research has also been extensively conducted to gain a deeper understanding, with fundamental findings such as the driving force of self-assembly and structuring [25,26]. Spectroscopic knowledge such as electron microscopy, X-ray analysis, and IR is extremely powerful for investigating structure formation in these studies. On the other hand, it is often difficult to obtain knowledge of the dynamics of water molecules due to the lack of broadband techniques in observation and analysis. 

Dielectric spectroscopy is one of the effective experimental methods to observe and analyze dynamic behaviors of water molecules and their hydrogen-bonding networks that exhibit the hierarchical structures of interacting molecules [27,28,29]. Polar water molecules have a high number density of dipole moments due to their small molecular size, so water contributes significantly to the dielectric constant for pure water and even aqueous mixtures. Dielectric spectroscopy is therefore one of the most suitable observational techniques for investigating the structure of water [27,30,31,32,33]. Due to differences in the dynamic behaviors of water molecules, various relaxation processes can be observed in different frequency regions, providing information on the restricted states of different water aggregates and dynamic properties. Because these relaxation processes overlap each other, it is difficult to analyze the dielectric relaxation curves using only measurements of a narrow frequency range. Measurements should be made over a wider frequency range to eliminate the effects of other processes. Broadband dielectric spectroscopy (BDS) measurements, which is a typical key technique in the research field of dielectric spectroscopy [28,29,34], solve these problems and allow the analysis of various water structures. In actual measurements, by designing and fabricating electrodes with different characteristics for each frequency range, we perform analysis suitable for relaxation processes distributed over a wide frequency region [28,34]. For these reasons, the recent BDS in particular is considered suitable for studying supramolecular gels with hierarchical structures over a wide region of length and time scales.

**Figure 2:**
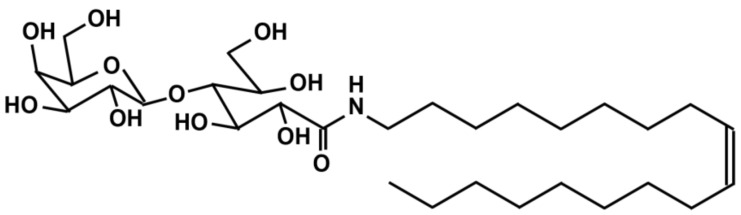
Chemical structure of N-oleyl lactobionamide.

N-oleyl lactobionamide is a low-molecular-weight hydrogelator with the chemical structure shown in Figure 2. The 5 wt% aqueous mixtures show a sol–gel transition at around 40 °C in the cooling process from 90 °C, as judged visually. Thus, the mixtures take a gel phase at lower temperatures, and the sol–gel transition is reversible in the heating and cooling processes. It is reported that the hydrogels change water into hydrogel by getting intertwined for self-assembly [10]. However, the structure formation mechanism of N-oleyl lactobionamide/water mixtures with gelation should be verified from the viewpoint of molecular dynamics. Though various works on analysis of mechanical properties and structures using microscope for low-molecular-weight hydrogelator/water mixtures have been reported so far, the structure formation mechanism has not been enough verified yet from the viewpoint of dynamic behaviors of molecules.

Therefore, in this work, we performed BDS measurements in order to verify the molecular mechanism of the sol–gel transition by the viewpoint of molecular dynamics. The molecular mechanism of the structural formation of fibrous supramolecular gel prepared by 5 wt% N-oleyl lactobionamide/water mixture was elucidated using the BDS measurements. Though this sample itself has not been used much, it is very convenient for the present dielectric study, since pure water can be used as solvent instead of organic solvents or solvent mixtures and the electric conductivity is not large compared with usual aqueous systems. The dynamic behavior of gelation observed for solutes and water molecules indicates a hierarchical structure formation process over various time scales and is expected to elucidate the molecular mechanism of gelation and its precursors.

## 2. Results and Discussion

### 2.1. Determination of the Sol–Gel Transition Temperature

**Figure 3:**
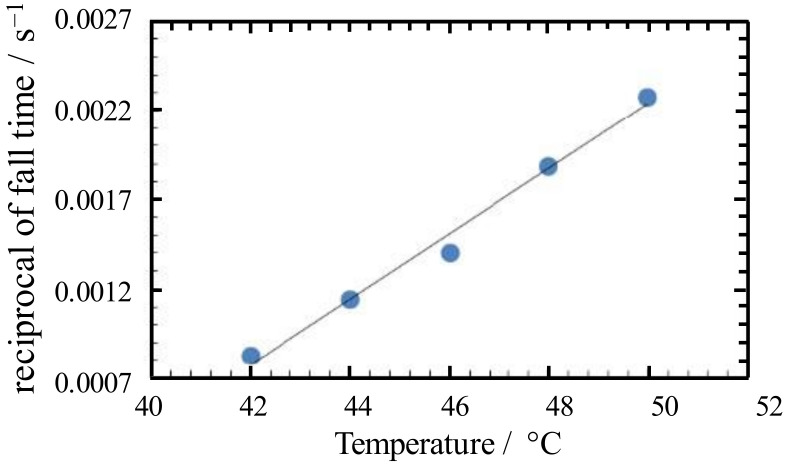
Temperature dependence of the reciprocal of the fall time for descent of the ball in 5 wt% N-oleyl lactobion/water mixture. The solid line indicates a linear relationship between the experimental plot (●) and temperature.

Figure 3 shows the temperature dependence of the reciprocal drop time of the ball descent in a 5 wt% N-oleyl lactobione/water mixture. In the sol state, the closer to the sol–gel transition temperature, *T*_gel_, from the high temperature side, the longer it takes for the ball to fall, and the reciprocal of the time seems to decrease linearly with temperature. The sol–gel transition temperature is thus defined as the temperature obtained by taking the inverse of the fall time as a function of temperature and extrapolating to zero [35]. Data acquisition conditions for extrapolation are highly dependent on the viscosity and modulus of the sample and require trial and error. The sol–gel transition of the N-oleyl lactobionamide/water mixture is reversible with temperature and exhibits a gel phase below the transition temperature, *T*_gel_ = 37.8 °C 

### 2.2. Dielectric Relaxation Curves

**Figure 4:**
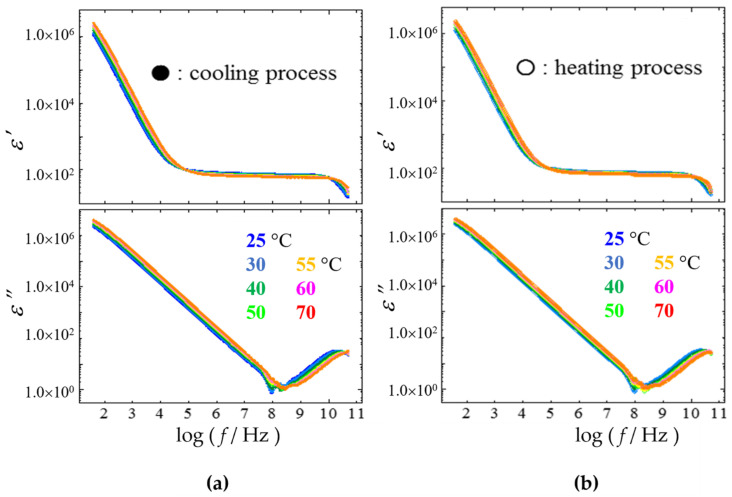
Frequency dependences of the real and imaginary parts of the complex permittivity for 5 wt% N-oleyl lactobionamide/water mixtures at various temperatures from 25 °C to 70 °C in the cooling [left, (**a**)] and heating processes [right, (**b**)]. The plot colors correspond to temperatures indicated in the figure.

Figure 4 shows plots of logarithmic frequency dependences of the real and imaginary parts of the complex permittivity obtained for N-oleyl lactobionamide/water mixtures at temperatures between 25 °C and 70 °C in the cooling and heating processes. The relaxation curves obtained in both processes show the reversible property of the transition. We observed a relaxation process attributed to free water around 20 GHz. In this paper, the relaxation process observed in the 10 GHz region is expressed as *h*-process. On the lower frequency side of the curves, a large relaxation process attributed to electrode polarization (EP) contributions by impurity ions in the mixture are clearly shown below 100 Hz [28,36]. In this paper, we call it the *l*_2_-process. For a more precise characterization of the dielectric properties, curve-fitting procedures of the contributions of individual relaxation processes to the relaxation curve were performed using the following equations:

(1) $$\varepsilon^{*}=\varepsilon^{\prime }-j\varepsilon^{\prime \prime }=\varepsilon_{\infty }+\overset{h,\;m,\;l}{\underset{k}{\sum }}\frac{\Delta \varepsilon_{k}}{1+{\left(j\omega \tau_{k}\right)}^{\beta_{k}}}+\frac{\Delta \varepsilon_{k}}{1+j\omega \tau_{l2}}+\frac{\sigma }{j\omega \varepsilon_{0}}$$

where ε* is the complex dielectric permittivity, *ω* is the angular frequency, *ε*_∞_ is the high frequency limit of the dielectric constant, ∆*ε* is the relaxation strength, *j* is the imaginary unit, *τ* is the relaxation time, *β* is relaxation time distribution parameter (0 < *β* ≤ 1), *σ* is the direct current (dc) conductivity and ε_0_ is the dielectric constant of vacuum. Equation (1) includes three Cole–Cole relaxation functions [37] for *h*-process, *m*-process and *l*_1_-process, and one Debye equation (*β* = 1) for the *l*_2_-process, and a dc conductivity term. 

**Figure 5:**
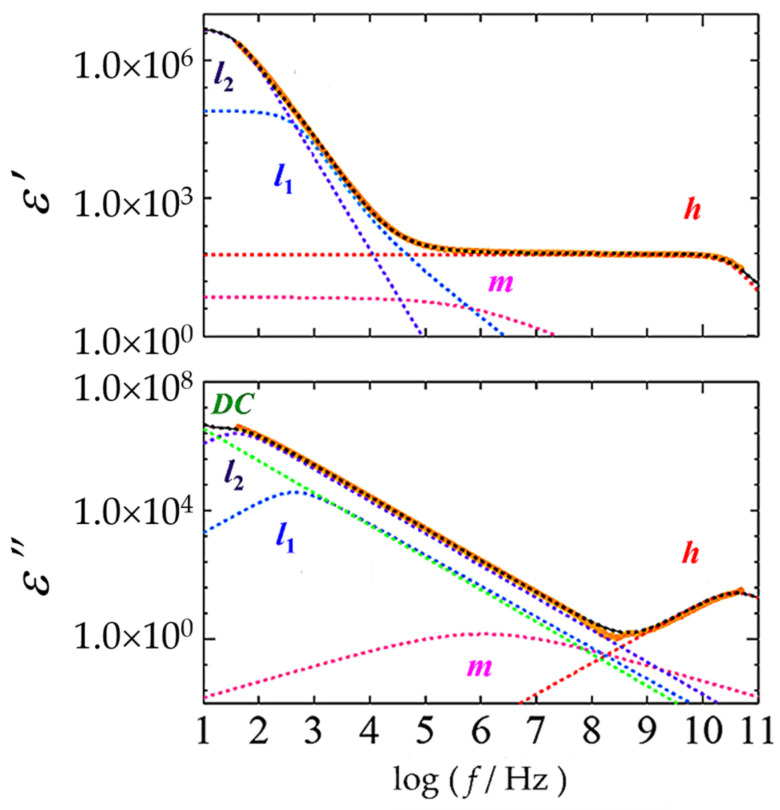
Dielectric relaxation curve (thick orange solid line) of 5 wt% N-oleyl lactobionamide/water mixture at 70 °C and each relaxation process obtained by the fitting procedures with Equation (1). Dotted lines represent *h-*(red), *m*-(magenta), *l*_1_-(blue) and *l*_2_-(purple) processes with the dc conductivity contribution (green) and calculated total (black).

Figure 5 shows a typical frequency dependence of complex permittivity for 5 wt% N-oleyl lactobionamide/water mixture at 70 °C with the curves given by Equation (1). Though *m*-process, *l*_1_-process and dc conductivity term were not clearly observed in the relaxation curve, these processes were necessary for curve-fitting. It should be also noted that the relaxation curve and processes shown in Figure 5 are similar to results obtained for previously reported liposome suspensions of 1,2-dipalmitoylsn-glycero-3-phosphacholine (DPPC) [38,39], and are important in the subsequent relaxation parameter analysis.

### 2.3. Relaxation Parameter Analysis

#### 2.3.1. *h*-Process

**Figure 6:**
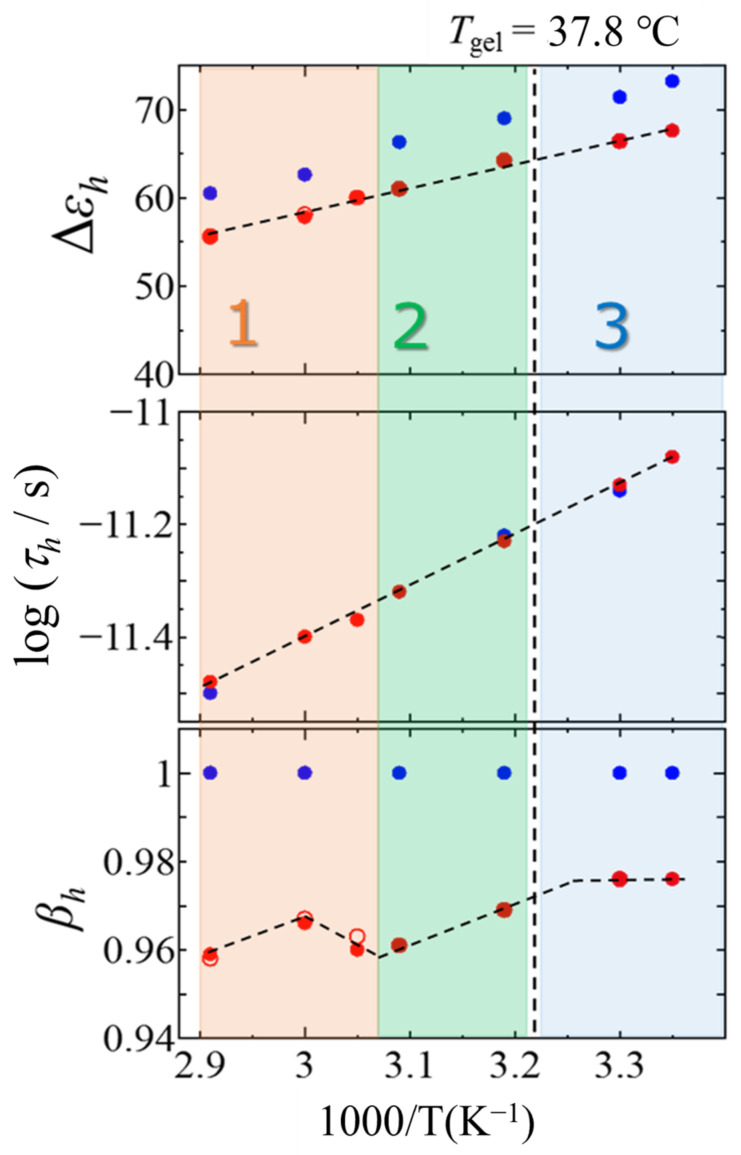
Temperature dependences of ∆*ε_h_*, *τ_h_*, *β_h_.* dielectric relaxation parameters for the *h*-process (●, ○) and those for ultrapure water (●). The closed and open symbols indicate cooling and heating processes, respectively. The vertical dashed lines indicate the sol–gel transition temperature, 37.8 °C. Numbers 1–3 in the figure indicate temperature regions. Temperature regions 1 and 2 are indicated by different colors as the higher and lower temperature regions of 52.5 °C where the relaxation parameter changes, and region 3 is the gel state temperature region below the sol–gel transition temperature.

Figure 6 shows the temperature dependence of ∆*ε_h_*, *τ_h_*, *β_h_* and relaxation parameters for pure water. The values of relaxation strength ∆*ε_h_* and relaxation time *τ_h_* showed general tendencies of increasing with decreasing temperature from 70 °C to 25 °C. The smaller ∆*ε_h_* values for the mixture comparing with those for pure water means smaller number density of water molecules. The values of relaxation time for *h*-process of N-oleyl lactobionamide/water mixtures and those for ultrapure water were not significantly different due to the dilute solution of 5 wt%. Considering the average characteristic time of rotational diffusion of water molecules, it is suggested the presence of N-oleyl lactobionamide molecule in water without preventing dynamic behaviors of the free water. This property is even more pronounced in dispersion systems in which solute molecules are associated [40].

The *β* value was unity for pure water at any temperatures, however, the values obtained for the mixture were smaller at any temperatures and show complicated temperature dependence. The smaller *β_h_* values suggest that the degree of heterogeneity in dynamic behaviors of water molecules is larger in the mixture because of interacting N-oleyl lactobionamide. It has already been reported in the case of DPPC liposomes [38,39] that the fluctuation of the *h*-process for surrounding water molecules increases with increasing mobility of the hydrophilic head group of the DPPC molecules. The temperature dependence of *β_h_* changes significantly around the sol–gel transition temperature of 37.8 °C and also around 52.5 °C in the sol phase. Therefore, as shown in Figure 6, we tentatively divided the temperature range into 1, 2 and 3 at those temperatures and colored each temperature region for clarity. The temperature dependence of *β_h_* changes significantly around the sol–gel transition.

The overall temperature dependence of the *β_h_* value shown in Figure 6 shows lower values at higher temperatures and higher values at lower temperatures. In contrast to this general trend, in temperature range 2, the *β_h_* values decrease with decreasing temperature on the cold side of temperature range 1 in the sol state. After increasing again, it shifts to temperature range 3 in the gel state and shows a constant value. The tendency for *β_h_* values to rise throughout the cooling process is due to the progressive self-assembly of individual N-oleyl lactobionamide molecules, which reduces the influence of surrounding water molecules on the dynamics via hydrogen bonding. It is thought that the fluctuation of the water structure decreases and the *β_h_* value increases. This result is in good agreement with the macroscopic viscosity increase of the system suggested by the increasing relaxation time trend. If this corresponds to the progress of elongation of rod-like micelles, it is well explained that the effect on the *β_h_* value disappears and becomes constant when temperature region 3 is entered beyond the macroscopic gelation temperature at which the fibers crosslink. The decrease in *β_h_* values seen during the cooling process from temperature range 1 to 2 is caused by the aggregation of N-oleyl lactobionamide molecules with reduced influence on the dynamics of surrounding water molecules. The trend is the opposite behavior. This is thought to be due to the fact that the *β_h_* value decreased due to the fluctuation of the water structure that causes the *h*-process, which fluctuates greatly in the initial stage of aggregation. At this time, it is considered that the trade-off relationship between the effect of promoting and suppressing the fluctuation of water molecule dynamics due to the aggregation of N-oleyl lactobionamide molecules collapsed. Therefore, in the structure of water at high temperatures, the dynamics of the surrounding water molecules and the hydrogen-bonding network are affected by the change in the number density of small-scale aggregates due to the self-assembly of N-oleyl lactobionamide molecules. It is suggested that during the cooling process in temperature region 2, the rod-like micelles are further elongated into filaments, initiate cross-linking, and undergo macroscopic gelation at the sol–gel transition temperature. After gel formation, in the stable gel state in temperature region 3, it can be considered that the *β_h_* value in the *h*-process does not change significantly. From these results, Figure 6 suggests that this series of structural formations and their fluctuations are reflected in the fluctuations of water dynamics in the *h*-process.

The dielectric behavior of the relaxation parameter of the *h*-process shown in Figure 6 reflects the dynamics of the polar head group of the N-oleyl lactobionamide molecule, as shown in DPPC liposome [38,39]. This is quite reasonable for *h*-process, since the relaxation process reflects the dynamics of a hydrogen-bonding network involving water molecules and is affected by interactions with the head groups of the N-oleyl lactobionamide molecules. Therefore, as shown in Figure 1, dielectric relaxation measurements indicate that the N-oleyl lactobionamide molecules have already begun to self-assemble in the sol phase before gelling during the cooling process, leading to gelation.

#### 2.3.2. *m*-Process

**Figure 7:**
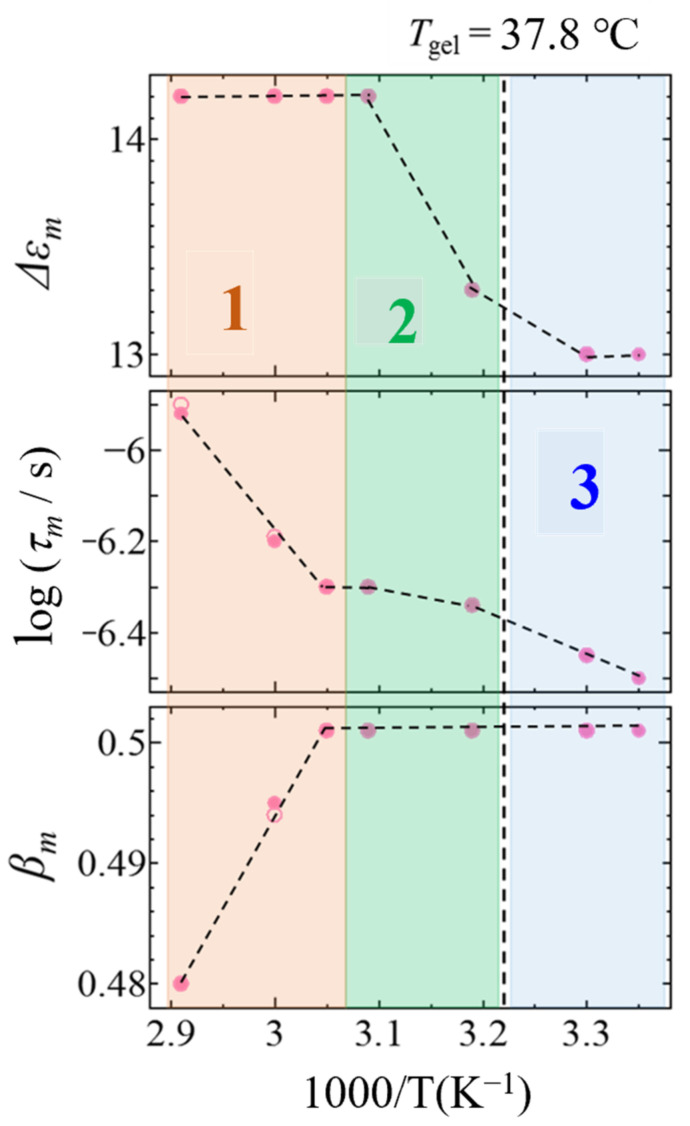
Temperature dependence of dielectric relaxation parameters, ∆*ε_m_*, *τ_m_* and *β_m_* for *m*-process. The closed and open symbols indicate cooling and heating processes, respectively. The vertical dashed lines indicate the sol–gel transition temperature, 37.8 °C. Numbers 1–3 in the figure indicate temperature regions. Temperature regions 1 and 2 are indicated by different colors as the higher and lower temperature regions of 52.5 °C where the relaxation parameter changes, and region 3 is the gel state temperature region below the sol–gel transition temperature.

Figure 7 shows temperature dependences of dielectric relaxation parameters, ∆*ε_m_*, *τ_m_* and *β_m_*. Our former works suggest that the *m*-process is a relaxation process attributed to dynamic behaviors of amphipathic molecules [33]. The ∆*ε_m_* values were larger and constant in temperature region 1, decrease with decreasing temperature in region 2; furthermore, the values take the same values in region 3. Since the value of ∆*ε* is proportional to the square of the dipole moment change and the number density causing *m*-process, the elongation of the rod micelles promotes the association of N-oleyl lactobionamide molecules, reduces their mobility and remains constant in the gel phase. These results confirm that *m*-process is due to dynamic behaviors of N-oleyl lactobionamide molecules.

The value of the relaxation time *τ_m_* decreased with decreasing temperature and showed different temperature dependence especially between temperature region 1 and regions 2 and 3. This result indicates that N-oleyl lactobionamide molecules have smaller scaled faster dynamics even in the rod micelle structures. The temperature dependence of the relaxation time of the dynamic behavior of amphiphilic molecules in aggregated structures decreasing with decreasing temperature has been reported as a ferroelectric property, even for DPPC liposome [39]. In addition, the value of the relaxation time distribution parameter βm increased with the decreasing temperature in temperature region 1, and remained constant at large values in temperature regions 2 and 3, showing a more uniform relaxation time distribution. This indicates that N-oleyl lactobionamide molecules are incorporated into a more uniform structure through cylindrical micelles and their fibrilization.

The temperature dependence of the relaxation parameter of the *m*-process shown in Figure 7 indicates a self-assembly process in which the N-oleyl lactobionamide molecules themselves are entrapped into rod micelles and restricted as shown in Figure 1. The experimental results of dielectric spectroscopy cannot prove the details of the aggregate structure and interaction of N-oleyl lactobionamide and water molecules. However, the BDS analysis is based on the dynamic structure of the constituent molecules to verify the validity of the hierarchical structure model shown in Figure 1, in which N-oleyl lactobionamide molecules self-assemble in the surrounding water molecules, grow into fibers and crosslink.

#### 2.3.3. *l*_1_-Process

**Figure 8:**
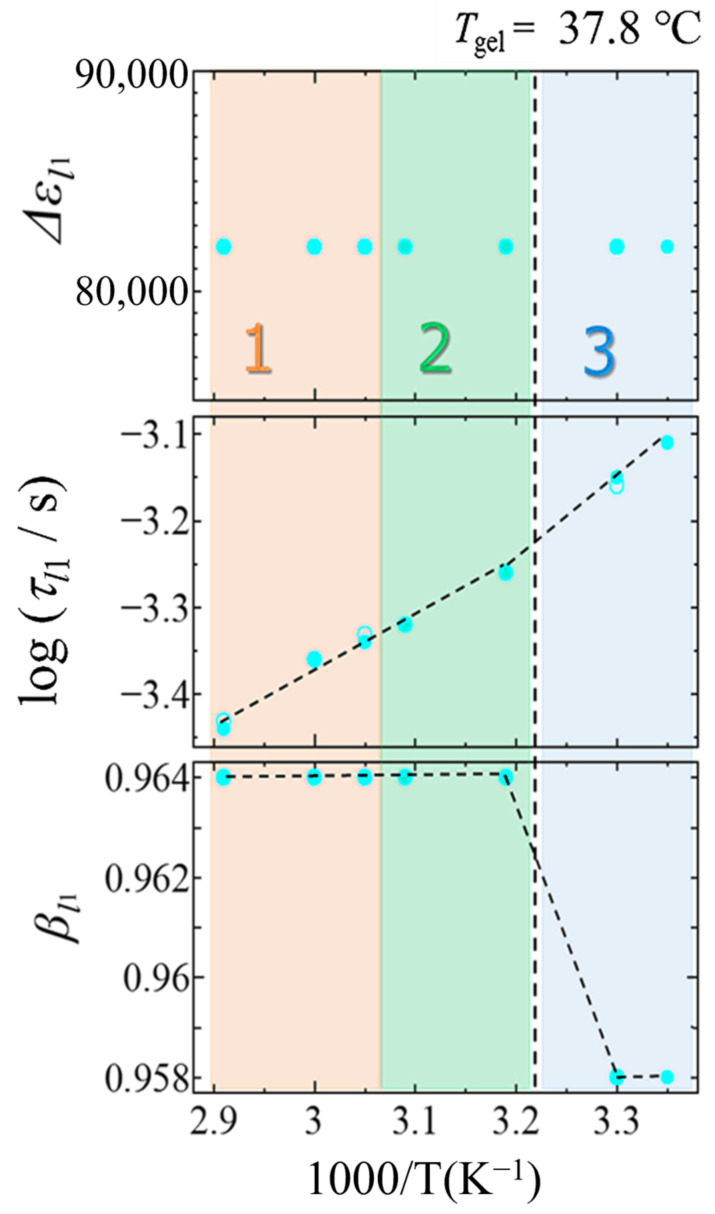
Temperature dependences of dielectric relaxation parameters, ∆*ε*, *τ* and *β* for the *l*_1_-process. The closed and open symbols indicate cooling and heating processes. The dashed lines indicate the sol–gel transition temperature. Numbers 1–3 in the figure indicate temperature regions. Temperature regions 1 and 2 are indicated by different colors as the higher and lower temperature regions of 52.5 °C where the relaxation parameter changes, and region 3 is the gel state temperature region below the sol–gel transition temperature.

Figure 8 shows temperature dependences of dielectric relaxation parameters, ∆*ε_l_*_1_, *τ_l_*_1_ and *β_l_*_1_. This relaxation process reflecting the ion dynamics in heterogeneous structures is usually observed as a contribution of the Maxwell–Wagner effect on the higher frequency side than the EP process due to the ion dynamics around the electrode structure.

The relaxation strength ∆*ε_l_*_1_ without clear temperature dependence over the entire temperature region in Figure 8 suggests that the number density of ions contributing to the *l*_1_ process does not change significantly, if the *l*_1_-process is due to the Maxwell–Wagner effect.

The relaxation time generally tends to increase with decreasing temperature. The temperature dependence of *τ_l_*_1_ slightly changes at the boundaries of the temperature regions. Temperature region 3 shows slightly larger slope, and the result suggests an increase in the energy required for ion migration in the gel state. In temperature region 3, a decrease in the *β_l_*_1_ value was also observed, indicating larger fluctuation for ion mobility in the gel state. The existence of local fluctuations of the local density of cross-linking points is generally recognized.

Considering that the *l*_1_-process reflects the contribution of the Maxwell–Wagner effect, the temperature dependence of the relaxation time and strength mentioned above can be explained by the existence of a specific interface where ions are bound in all temperature regions. However, it is difficult to imagine such a macroscopic structure formation occurs even in the sol state [41]. Then the *l*_1_ process, which was originally separated from the *l*_2_ process in this study, may be attributed to one of the complex behaviors exhibited by the EP process. It has been suggested that some EP processes may be triggered by ions forming layered structures around the electrodes [42]. Irrespective of these two relaxation mechanisms, the interpretation remains that the decrease in *β_l_*_1_ value indicates an increase in the heterogeneous structure of the gel phase.

The temperature dependence of the relaxation parameters of the *l*_1_-process, shown in Figure 8, could explain the large-scale structure formation associated with gelation model in Figure 1 by ionic behavior reflecting heterogeneous structures. The analytical technique using BDS method has the characteristics to investigate various water structures spread over a wide time and length scales in aqueous complex systems, such as supramolecular gels and hydrogels. The analysis and interpretation of each relaxation process performed in the present paper demonstrated its applicability to a wide range of aqueous systems in various fields and further developments expected in the future.

## 3. Conclusions

BDS measurements of the gelation process during cooling of a 5 wt% N-oleyl lactobionamide/water mixture were performed, and relaxation parameter analysis of the three observed relaxation processes was performed. It was found at around the sol–gel transition temperature (37.8 °C) and around 53 °C in the sol state. Fluctuations of the dynamic behavior of water evaluated by the relaxation time distribution parameter increased with each structure formation. Fluctuation of solute molecules caused the formation of rod-like micelle structure, which is the precursor of gelation, already started in the sol state around 53 °C and extended into filaments. Furthermore, large-scale structural perturbations showed macroscopic gelation at 37.8 °C due to three-dimensional network cross-linking. This study clearly demonstrated how relaxation parameter analysis by BDS is effective for understanding the details of the gelation mechanism.

## 4. Materials and Methods

### 4.1. Sample Preparations

The N-oleyl lactobionamide used in the present work is one of the low-molecular-weight hydrogelators. Measurement samples were prepared using N-oleyl lactobionamide prepared according to standard methods already disclosed in the literature [43,44,45,46]. N-oleyl lactobionamide thus obtained was first added to ultrapure water in a vial to prepare a 5% by weight N-oleyl lactobionamide/water mixture. The pure water was produced in a water purifier (Direct-Q^®^ UV3 Water Purification System, Merck Millipore Corporation, Darmstadt, Germany) and had a resistivity of 18.3 MΩ cm. The sample was heated and melted at 95 °C for 180 min using an aluminum block constant temperature bath (MG-1200, EYELA, Tokyo, Japan). In order to prevent moisture evaporation at higher temperatures, the cap of the vial tube was sealed with a dental impression material (Exahyflex injection type, GC Corporation, Tokyo, Japan) and left at room temperature of 19–20 °C for 24 h. The sample exhibited a sol–gel transition during cooling process.

### 4.2. Sol–Gel Transition Temperature Measurement

The sol–gel transition temperature was determined by ball-drop method [35] with a stainless ball (diameter: 2.0 mmϕ). The sample solution containing the stainless ball was gently mixed in a sealed glass tube at 90 °C, and then the glass tube was immersed in a water bath that had been controlled at the desired temperature with an uncertainty of ±0.05 °C. Before measuring the time of a descent of the ball in the sample, the glass tube was kept at a constant temperature for 40 min to ensure the thermal equilibrium of the solution. After measuring the time of descent of the stainless ball, the solution was stirred again at 90 °C before the measurement at the next temperature to eliminate the effect of the breakdown of the structures by the stainless ball. 

### 4.3. Dielectric Measurement

Dielectric measurements were performed in the frequency range from 40 Hz to 50 GHz using impedance analyzer (IA, Precision Impedance Analyzer 4294A, 40 Hz–110 MHz, Agilent Technologies, Santa Clara, CA, USA) and network analyzer (NA, PNA-L Microwave Network Analyzer N5230C, 100 MHz–50 GHz, Agilent Technologies, Santa Clara, CA, USA). A coaxial electrode (High-Temperature Dielectric Probe 8570C, Agilent Technologies, Santa Clara, CA, USA) with an inconel outer conductor (outer diameter: 19.0 mmϕ), a stainless-steel inner conductor (diameter: 1.0 mmϕ), and a glass insulator was employed for IA measurements. A coaxial probe (Performance Probe 85070E, Agilent Technologies, Santa Clara, CA, USA) with a stainless-steel outer conductor (outer diameter: 9.5 mmϕ) and a nickel-plated tungsten inner conductor (diameter: 1.6 mmϕ) sealed with borosilicate glass at the tip was employed for NA measurements. 

The dielectric measurements were performed in the temperature range from 25 °C to 70 °C and in the cooling process and heating process. Sample temperature was controlled by thermostatic bath with an accuracy of ±0.05 °C. Dielectric measurements were performed after the sample was left in thermal equilibrium for 30 min at each measurement temperature.

## Data Availability

Not applicable.
